# Reduced Sarcolemmal Membrane Repair Exacerbates Striated Muscle Pathology in a Mouse Model of Duchenne Muscular Dystrophy

**DOI:** 10.3390/cells11091417

**Published:** 2022-04-22

**Authors:** Brian J. Paleo, Kevin E. McElhanon, Hannah R. Bulgart, Kassidy K. Banford, Eric X Beck, Kristina M. Sattler, Briana N. Goines, Shelby L. Ratcliff, Kelly E. Crowe, Noah Weisleder

**Affiliations:** 1 Department of Physiology and Cell Biology, Davis Heart and Lung Research Institute, The Ohio State University, Columbus, OH 43210, USA; paleo.1@buckeyemail.osu.edu (B.J.P.); mcelhanon.1@buckeyemail.osu.edu (K.E.M.); hannah.bulgart@osumc.edu (H.R.B.); kassidy.banford@osumc.edu (K.K.B.); exbeck@gmail.com (E.X.B.); 2 Department of Biology, School of Behavioral & Natural Sciences, Mount St. Joseph University, Cincinnati, OH 45233, USA; sattler.78@buckeyemail.osu.edu (K.M.S.); briana.goines@msj.edu (B.N.G.); shelby.ratcliff@msj.edu (S.L.R.); kelly.crowe@msj.edu (K.E.C.)

**Keywords:** dystrophy, membrane repair, muscle, fibrosis, sarcolemma

## Abstract

Duchenne muscular dystrophy (DMD) is a common X-linked degenerative muscle disorder that involves mutations in the DMD gene that frequently reduce the expression of the dystrophin protein, compromising the structural integrity of the sarcolemmal membrane and leaving it vulnerable to injury during cycles of muscle contraction and relaxation. This results in an increased frequency of sarcolemma disruptions that can compromise the barrier function of the membrane and lead to death of the myocyte. Sarcolemmal membrane repair processes can potentially compensate for increased membrane disruptions in DMD myocytes. Previous studies demonstrated that TRIM72, a muscle-enriched tripartite motif (TRIM) family protein also known as mitsugumin 53 (MG53), is a component of the cell membrane repair machinery in striated muscle. To test the importance of membrane repair in striated muscle in compensating for the membrane fragility in DMD, we crossed TRIM72/MG53 knockout mice into the *mdx* mouse model of DMD. These double knockout (DKO) mice showed compromised sarcolemmal membrane integrity compared to *mdx* mice, as measured by immunoglobulin G staining and ex vivo muscle laser microscopy wounding assays. We also found a significant decrease in muscle ex vivo contractile function as compared to *mdx* mice at both 6 weeks and 1.5 years of age. As the DKO mice aged, they developed more extensive fibrosis in skeletal muscles compared to *mdx*. Our findings indicate that TRIM72/MG53-mediated membrane repair can partially compensate for the sarcolemmal fragility associated with DMD and that the loss of membrane repair results in increased pathology in the DKO mice.

## 1. Introduction

Plasma membrane disruptions occur in many cell types in a variety of tissues as the result of cellular injury, such as physical damage or oxidative stress. Once the plasma membrane is physically disrupted, the cell uses a specialized membrane repair process to reseal the injury and restore the barrier function of the membrane to allow for survival of the cell. This process is triggered by the influx of extracellular calcium into the cell through membrane disruptions; this leads to the recruitment of intracellular vesicles and associated proteins that create a membrane repair patch to reseal the injury [1,2,3,4]. Although exocytosis [1,5,6] and endocytosis [7] of these intracellular vesicles are thought to be the predominant processes used to reseal the sarcolemmal membrane, other cellular mechanisms have been shown to contribute to membrane repair in a cell type- and damage-dependent manner [8,9,10,11].

Membrane repair is particularly essential in striated muscle myocytes, as the contractile nature of these cells exposes the sarcolemmal membrane to increased mechanical stress [3,12]. Several proteins that function in vesicle trafficking [13,14,15] or fusion [16] contribute to the sarcolemmal membrane repair process. TRIM72/MG53 is an E3 ubiquitin ligase that is part of the tripartite motif (TRIM)/RING B-box Coiled Coil (RBCC) family of proteins [17,18]. It was first identified as mitsugumin 53 (MG53) through an immunoproteomic screen looking for proteins enriched in the triad junction of skeletal muscle [19] and was later designated TRIM72. While TRIM72/MG53 functions in membrane repair, it has also been shown to contribute to other processes, including whole-body metabolism [20,21], in other recent studies. Multiple lines of evidence support that TRIM72/MG53 is an important component of the membrane repair process, including studies showing increased membrane repair when TRIM72/MG53 is overexpressed and that a TRIM72/MG53 knockout mouse displays defective membrane repair and a progressive myopathy [22,23,24,25].

TRIM72/MG53 regulates vesicle trafficking and exocytosis [26], which leads to its role in the membrane repair process [14,22]. Several other proteins, including dysferlin [27,28,29], annexins [29,30,31,32,33,34], and caveolins [35,36], have also been linked with sarcolemma repair, with many of these proteins interacting with each other in the context of membrane repair, leading up to the formation of a protein-enriched cap associated with the repair patch [31]. For example, TRIM72/MG53, caveolin-3, and dysferlin all form a protein complex that facilitates membrane repair [15]. These membrane repair proteins have been shown to contribute to human disease; disruption of the TRIM72/MG53, caveolin-3, and dysferlin complex, as well as mutations in the dysferlin [27,37,38,39,40] and caveolin-3 [41,42] genes, can result in muscular dystrophies. TRIM72/MG53 has also been linked with multiple pathologies, including muscular dystrophies [15,43].

TRIM72/MG53 and other proteins of the membrane repair machinery are thought to support tissue homeostasis, both by maintaining cellular function and preventing energetically demanding replacement following cell death. During normal physiology, membrane repair can play a limited role in some tissues; however, it becomes more important during stress. When cell membrane disruptions are more prevalent, including during injuries induced by permeabilizing toxins [28,44], ischemia reperfusion events [45,46,47,48], or muscular dystrophies [27,49], there is an increased need for membrane repair.

Muscular dystrophies are a group of disorders characterized by loss of muscle mass and muscle weakness. Duchenne muscular dystrophy (DMD) is the most common form of muscular dystrophy, with a prevalence in the United States and Europe of approximately 6 per 100,000 individuals [50,51]. DMD is caused by mutations in the DMD gene, which encodes a protein that links the extracellular matrix and the intercellular cytoskeleton, supporting the structure and stability of the sarcolemmal membrane. As this membrane in DMD patients is more fragile than unaffected muscle, the lack of dystrophin leads to greater damage to both skeletal myocytes and cardiomyocytes during normal body movement. It is likely that this extensive muscle membrane damage requires increased activation of membrane repair to compensate for the fragility of the membrane. Not surprisingly, many membrane repair proteins are upregulated in patients with DMD [43], and increasing membrane resealing has been suggested as a strategy for treatment of the disease. In fact, this strategy has been effective in the *mdx* mouse model, in which treatment with recombinant human MG53 protein (rhMG53) [52] or poloxamer 188 (P188) [53,54] to increase membrane resealing decreased several structural and functional hallmarks of the disease. While there have been previous assessments of the status of the membrane repair response in dystrophic muscle [55,56,57,58,59], the full extent to which membrane repair contributes to slowing the progression of DMD remains unclear.

Previous studies have attempted to address this question by knocking out the expression of specific membrane repair proteins in the *mdx* mouse background. When dysferlin knockout mice were crossed with dystrophin-deficient mice, the pathology was increased in the double knockout mouse in an age-dependent manner [60]. This study focused mainly on skeletal muscle and it showed the importance of maintaining dysferlin function to compensate for the loss of dystrophin in the *mdx* mouse. Given that TRIM72/MG53 binds dysferlin [15,61] and regulates the translocation of dysferlin to sites of membrane disruption [61], a question remains about the importance of TRIM72/MG53 in particular, and membrane repair in general, in the progression of DMD. In this study, we explore the extent to which TRIM72/MG53 can compensate for the extensive membrane injury in the *mdx* mouse. To do so, we crossed the TRIM72/MG53 knockout mouse into the *mdx* mouse model of DMD to create a double knockout (DKO) mouse for TRIM72/MG53 and dystrophin. This mouse model shows reduced sarcolemmal membrane repair capacity and age-dependent increases in the hallmarks of DMD, including decreased force production by the extensor digitorum longus (EDL) muscle and increased fibrosis in multiple skeletal muscles.

## 2. Materials and Methods

### 2.1. Mouse Model Breeding

Mice were housed and bred at standard conditions with a temperature of 22 ± 2C and a 12-h/12-h light cycle. Mice were provided standard mouse chow and drinking water ad libitum. All animal care and experimental procedures were preapproved by The Ohio State University Institutional Animal Care and Use Committee. *TRIM72* knockout mice (*TRIM72^−^/^−^*) were previously generated [22] and crossed with commercially available *mdx* mice (Jackson Laboratory). Three generations of crosses of male progeny with *mdx* female mice produced littermates *TRIM72^−^/^−^*/*mdx* double knockout (DKO), or *TRIM72^+^/^+^*/*mdx* (*mdx*). Lack of MG53 and dystrophin expression was confirmed by Western blotting using standard techniques as described below.

### 2.2. Tissue Preparation for Histological Procedures

Male mice at various ages (indicated where appropriate) were euthanized via CO_2_ asphyxiation and cervical dislocation. Hind limb muscles were dissected and fixed for 24 h with 10% phosphate-buffered formalin, followed by a 70% ethanol incubation for 24 h. Tissue was then processed and embedded in paraffin (Thermo Scientific, Waltham, MA, USA) and 12 μm sections were collected on SuperFrost Plus slides (Fisher Scientific, Hampton, NH, USA). Slides were deparaffinized with two xylene incubations and rehydrated with a series of ethanol incubations at the following concentrations: 100%, 100%, 95%, 70%, 50%. The slides were then stained for hematoxylin/eosin (Thermo Scientific, Waltham, MA, USA) or Masson’s trichrome (American MasterTech, Lodi, CA, USA) for immunohistochemical staining and analysis.

### 2.3. Myocyte Size Measurements

Myocyte cross-sectional area (CSA) was determined from sections of EDL, soleus, and TA muscles stained with hematoxylin/eosin. Image files were blinded before analysis to preclude any bias. Myocyte CSA was determined by outlining individual myocytes using ImageJ. Myocyte CSA was analyzed for their frequency distribution (200 µm bins), and graphed based on the percentage of myocytes corresponding to the binned myocyte size.

### 2.4. Central Nuclei Measurements

Centrally located nuclei were counted from myocytes measured for CSA. Myocytes were counted as positive for centrally located nuclei if nuclei were located inside of the perimeter of the outlined myocyte. Image files were blinded before analysis to preclude any bias.

### 2.5. IgG Staining and Analysis

First, 12 μm paraffin-embedded sections of EDL, soleus, and TA muscles were deparaffinized with xylene incubations and rehydrated with ethanol incubations, followed by antigen retrieval with Citra Plus Solution (Biogenex, Fremont, CA, USA). Tissue sections were blocked with 2.5% bovine serum albumin (Sigma Aldrich, St. Louis, MO, USA) for 1 h at room temperature, and incubated overnight at 4C with goat anti-mouse IgG antibody conjugated with Alexa Fluor 488 (Life Technologies, Carlsbad, CA, USA). ImageJ was utilized to analyze the IgG positivity within the muscle tissue. To eliminate dark background without tissue present and staining artifacts (including areas with tissue folding or separation due to processing and heavily fibrotic or necrotic areas where distinct fibers with intact membranes could not be identified), the outline of the tissue was traced to only include analysis of the skeletal muscle tissue. The IgG-positive area was divided by the total area to determine the percent area of IgG-positive skeletal muscle per image. To preclude bias in the selection of areas, all experiments were performed in a blinded fashion.

### 2.6. Masson’s Trichrome Analysis

Analysis of the percent area of fibrosis was conducted using ImageJ (NIH, Bethesda, MD, USA) on Masson’s trichrome-stained sections. First, 20× images were stitched together, encompassing the entire muscle, using the EVOS FL Auto 2 inverted microscope imaging system (ThermoFisher, Waltham, MA, USA). ImageJ was utilized to identify the skeletal muscle area within each stitched image. To exclude histological artifacts (including areas with tissue folding or separation due to processing), the ImageJ plug-in color deconvolution was used to separate the blue channel and an additional threshold corresponding to areas of the tissue positive for fibrosis was measured. The percent area of fibrosis in the tissue section was determined by dividing the fibrosis-positive tissue area by the total tissue area of each image representing whole tissue. To preclude bias in the selection of areas, all experiments were performed in a blinded fashion.

### 2.7. Ex Vivo Assessment of Skeletal Muscle Contractility 

Isolated mouse muscle contractility was measured as described previously [22]. Briefly, EDL and soleus muscles were isolated and mounted between two electrodes in a chamber filled with Tyrode’s solution supplemented with 2 mM Ca and 12 mM glucose. Oxygen was bubbled in the Tyrode’s solution to ensure sufficient oxygenation of the muscles during the protocol. Muscles were stretched to ensure maximal force using 80 Hz pulses, and a constant stimulatory voltage (one 80 Hz pulse every minute for 30 min) was applied to allow the muscles to equilibrate. Following equilibration, muscles were stimulated at frequencies from 1 to 150 Hz to generate a force vs. frequency curve. The force versus frequency curve was generated by stimulating muscles at the following frequencies: 1, 5, 10, 20, 30, 40, 50, 60, 80, 100, 120, 140, and 150 Hz. Maximal force (F-max) was taken from the peak force on the resulting force frequency curve.

### 2.8. Western Blotting and ELISA Measurements

Skeletal muscle tissue was dissected from mice and protein lysate was isolated using Radioimmunoprecipitation Assay buffer (RIPA; Cell Signaling Technology, Danvers, MA, USA). Protein concentrations were determined by the standard Bradford Assay using bovine serum albumin (BSA) standards ranging from 0 to 1 mg/mL. Then, 20 μg protein samples were separated by SDS-PAGE at room temperature on 4–15% gradient gels (Bio-Rad, Hercules, CA, USA) and were transferred on ice to 0.45 μm nitrocellulose membranes (Bio-Rad, Hercules, CA, USA). Membranes were stained with Ponceau S stain (Millipore Sigma, Burlington, MA, USA) and then photographed for determination of protein loading levels. Membranes were probed for TRIM72/MG53 with a custom polyclonal antibody (Pacific Immunology, San Diego, CA, USA), dysferlin (Leica Biosystems, Deer Park, IL, USA), caveolin-3 (Abcam, Cambridge, UK), anti-mouse horseradish peroxidase (HRP)-conjugated secondary antibodies, and anti-rabbit HRP-conjugated secondary antibodies (Cell Signaling Technology, Danvers, MA, USA). The blots were developed using enhanced chemiluminescence (ECL) substrate (Bio-Rad, Hercules, CA, USA). An Azure Biosystems imager was used to visualize chemiluminescent blots. Quantification of immunoreactive bands was performed using ImageJ’s integrated density measurement and normalized to total protein levels using Ponceau S staining. Creatine kinase (CK) levels were measured in mouse serum using a mouse CK enzyme-linked immunoassay (ELISA) kit (Novus Biologicals, Centennial, CO, USA) per manufacturer’s directions.

### 2.9. Membrane Repair Assessment following Laser Injury

Whole flexor digitorum brevis (FDB) muscles were dissected from mice and mounted on a 35 mm glass-bottom imaging dish with commercially available liquid bandage. Physiologic Tyrode’s solution containing 2.0 mM Ca^2+^ was added to the dish to induce membrane damage. The Olympus FV1000 multi-photon laser scanning confocal system was used to irradiate the sarcolemma to assess membrane repair. Membrane injury was induced in the presence of 2.5 μM FM4-64 fluorescent lipophilic dye (Life Technologies, Carlsbad, CA, USA). A circular area was selected along the edge of the cell membrane and irradiated at 20–30% laser power for 5 s (s). Pre- and post-damage images were captured every 3 s, continuing for 57 s. The ImageJ software (NIH, Bethesda, MD, USA) was used to analyze cell membrane repair kinetics by measuring the fluorescence intensity, encompassing the site of damage and the background dye fluorescence. ΔF/F0 values were calculated with the following equation at each timepoint: (injury fluorescence-background fluorescence)/background fluorescence. To preclude any potential for bias, all experiments were performed in a blinded fashion.

### 2.10. Statistical Analysis

Graphical representation and statistical analysis of data was performed using Prism version 8 (GraphPad, San Diego, CA, USA). All results are presented as mean ± SEM. Cross-sectional area measurements were grouped based on frequency distribution in bins of 200 µm. Measurements were analyzed by a two-way ANOVA with a Bonferroni post-hoc test. Central nuclei, F-max, laser injury AUC, percent fibrosis, CK, and protein expression measurements were analyzed by unpaired two-tailed *t*-test assuming unequal variances, and with Welch’s *t*-test for unbalanced designs. Other appropriate statistical tests, including Mann–Whitney tests, were used on specific data sets as indicated in the text.

## 3. Results

### 3.1. Membrane Repair Is Compromised in the Six-Week-Old DKO Mouse

**Figure 1:**
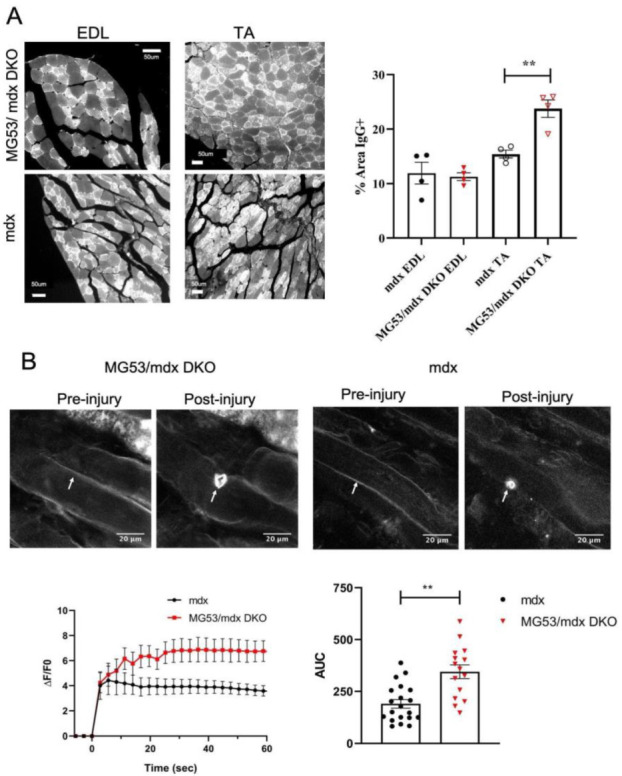
Membrane repair and integrity is compromised in DKO mice. (**A**) Representative images of IgG staining. Paraffin sections of EDL and TA muscles stained with fluorescent anti-mouse-IgG antibodies demonstrate the distribution of IgG-positive and IgG-negative myocytes in selected skeletal muscles. Quantification analysis of IgG-positive myocytes for the EDL and TA show a significant increase in positive muscle myocyte damage in the DKO group. EDL *n* = 4, *p* = 0.8456; TA *n* = 4, *p* = 0.0081. (**B**) Representative images of FM4-64 dye in whole FDB muscles from *mdx* and DKO mice. The area under the curve (AUC) of FM4-64 fluorescence traces displays different membrane resealing in DKO mice. *mdx n* = 22 myocytes; DKO *n* = 16 myocytes, *p* = 0.0052. ** = *p* < 0.01. Data represented as means ± SEM. (**A**) Scale bar = 50 µm, (**B**) Scale bar = 20 µm.

Membrane repair has been shown to be a crucial process to maintain the integrity of skeletal muscle, and targeting membrane repair has shown potential as a therapeutic approach in the *mdx* mouse [52]. Since the *mdx* mouse lacks functional dystrophin, the integrity of the plasma membrane is compromised, potentially leading to myocytes becoming more reliant on membrane repair to prevent the death of myocytes. To further investigate this reliance on membrane repair, we crossed the TRIM72/MG53 knockout mice into the *mdx* mouse model of DMD. These double knockout (DKO) mice were viable and, when sacrificed at 6 weeks of age, did not show differences in the wet weight of the heart, diaphragm, or skeletal muscle as compared to *mdx* mice at this age, even when normalized to body weight (*mdx*: 23.4 g ± 0.4, DKO: 24.0.9 g ± 0.6). We then assessed the extent of changes in sarcolemmal membrane permeability by immunostaining the EDL and tibialis anterior (TA) muscles from 6-week-old *mdx* and DKO mice to quantify IgG antibodies trapped in myocytes after membrane injury. DKO TA muscles showed a significant increase in IgG-positive myocytes as compared to *mdx* muscles (Figure 1A). To directly test membrane repair, whole flexor digitorum brevis (FDB) muscle taken from 6-week-old mice was subjected to infrared laser injury ex vivo using a multiphoton microscope. DKO mice showed a significant increase in dye influx, indicating a defect in the membrane repair response of these myocytes (Figure 1B). These findings indicate that although the *mdx* mouse has compromised membrane integrity, an additional defect in membrane repair due to a loss of TRIM72/MG53 can exacerbate this compromised membrane integrity. This is further supported by an increase in serum CK levels in the DKO mice (*mdx* at 112.8 ± 10.13 ng/μL, *n* = 10 vs. DKO at 162.4 ± 11.57 ng/μL, *n* = 6 with *p* = 0.011 by Mann–Whitney test). Thus, the DKO model provides an opportunity to evaluate the impact of membrane repair in compensating for the membrane fragility in DMD muscle and altering the pathologic hallmarks of this disease.

### 3.2. Compensatory Changes in Membrane Repair Proteins in the DKO Mouse Skeletal Muscle

**Figure 2:**
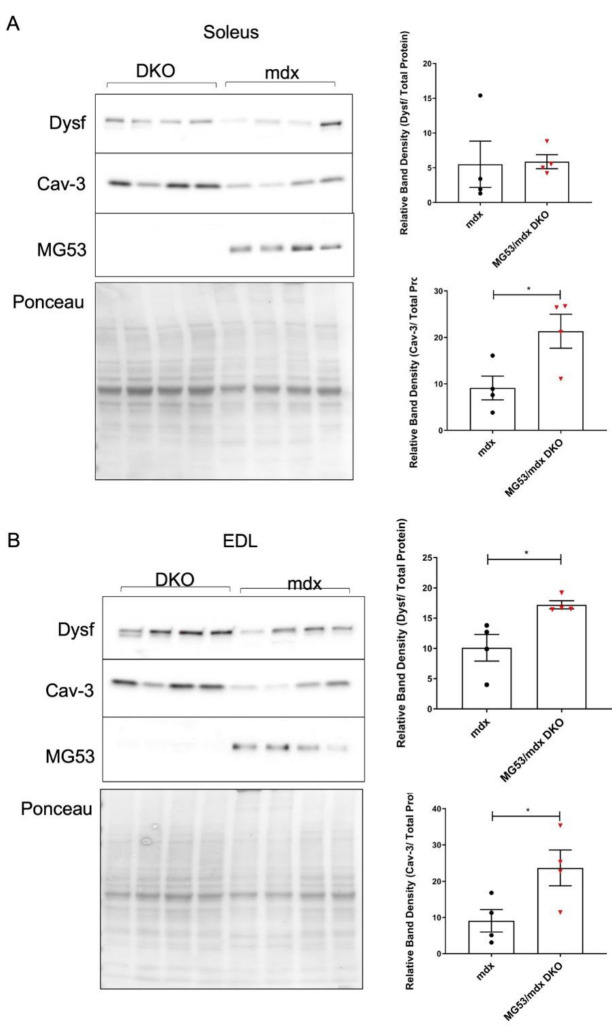
Membrane repair proteins are altered in DKO mice. (**A**) Soleus and (**B**) EDL muscle lysates from 6-week-old mice were used for Western blotting to detect changes in membrane repair proteins. Cav-3 protein expression increases in both muscles; while dysferlin levels are only elevated in EDL muscles. Statistical analysis was performed with an unpaired two-tailed *t*-test assuming unequal variances, and with Welch’s *t*-test for unbalanced designs. (**A**) (*n* = 4) Dysf *p* = 0.9177, Cav-3 *p* = 0.0339; (**B**) (*n* = 4) Dysf *p* = 0.0212, Cav-3 *p* = 0.0458. * = *p* < 0.05. Data represented as means ± SEM.

Our previous studies show that TRIM72/MG53 forms a functional complex with dysferlin and caveolin-3 [15]. While the absence of TRIM72/MG53 does compromise repair in the *mdx* background, it is not clear if there is compensation by increased expression of other membrane repair proteins. In fact, in both the EDL and soleus, we observed an increase in caveolin-3 protein. Interestingly, we only resolved increased dysferlin expression in the soleus after normalizing to Ponceau S staining of the membrane as a loading control (Figure 2). These changes in caveolin-3 and dysferlin levels are in excess of the already elevated levels of repair proteins seen in dystrophic muscle from human patients and the *mdx* mouse model [43].

### 3.3. Deletion of TRIM72/MG53 Does Not Alter Skeletal Muscle Histological Pathology in Six-Week-Old DKO Mice

**Figure 3:**
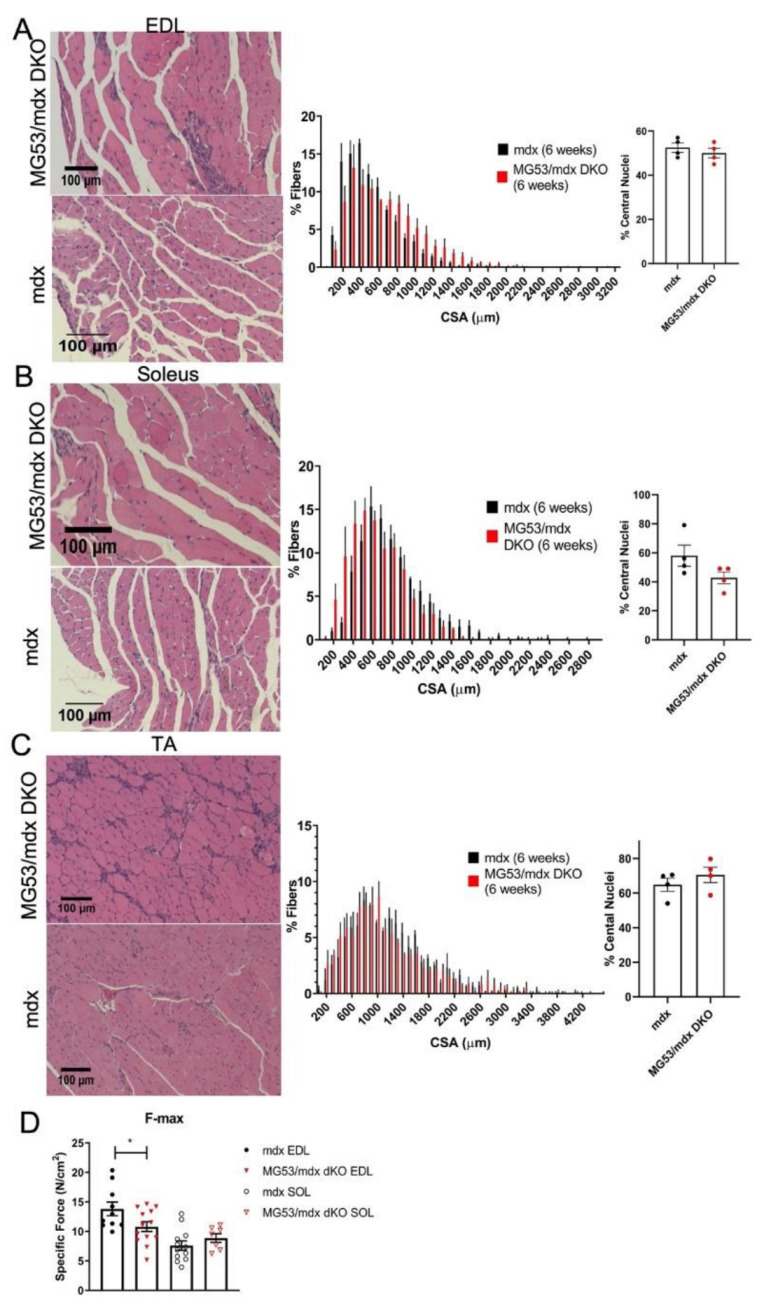
Histological analysis of skeletal muscle of *mdx* and DKO mice at 6 weeks. H&E staining of EDL (**A**), soleus (**B**), and tibialis anterior (**C**) sections from *mdx* and DKO mice at six weeks of age was analyzed for frequency distribution of myocyte cross-sectional area (CSA) and central nuclei. There was no difference in the histology of any of the muscles (*n* = 3) for all groups tested. (**D**) Maximal force (F-max) of ex vivo EDL and soleus muscles from *mdx* and DKO mice. Force was significantly reduced in the DKO EDL muscles. Differences in F-max were compared by unpaired two-tailed *t*-test assuming unequal variances, and with Welch’s *t*-test for unbalanced designs. F-max EDL: *mdx n* = 10, DKO *n* = 13 *p* = 0.0414; Soleus: *mdx n* = 12, DKO *n* = 7, *p* = 0.2532. * = *p* < 0.05. Data represented as means ± SEM to indicate the confidence level in the mean at each frequency. Scale bar = 100 µm.

The *mdx* mouse has a discernable histopathology at 3–5 weeks of age, including extensive muscle damage, inflammation, and subsequent regeneration [62,63]. Histopathological analysis of various muscles showed that there were no significant differences observed in cross-sectional area or central nuclei count in the EDL, soleus, and TA muscles of the *mdx* and DKO mice (Figure 3A–C). However, the DKO mice showed a significant decrease in maximal contractile force in the EDL when compared to the *mdx* mouse (Figure 3D). There was no observed difference between the force outputs of soleus muscles between the two genotypes (Figure 3D). As our results indicate decreased EDL maximal contractile force but no difference in cross-sectional area (CSA), TRIM72/MG53 may play a role in maintaining the level of force produced by this predominantly fast twitch muscle, potentially by affecting the early stages of muscle regeneration and subsequent myocyte maturation [64,65].

### 3.4. Decreased Skeletal Muscle Force Production in Aged DKO Mice

**Figure 4:**
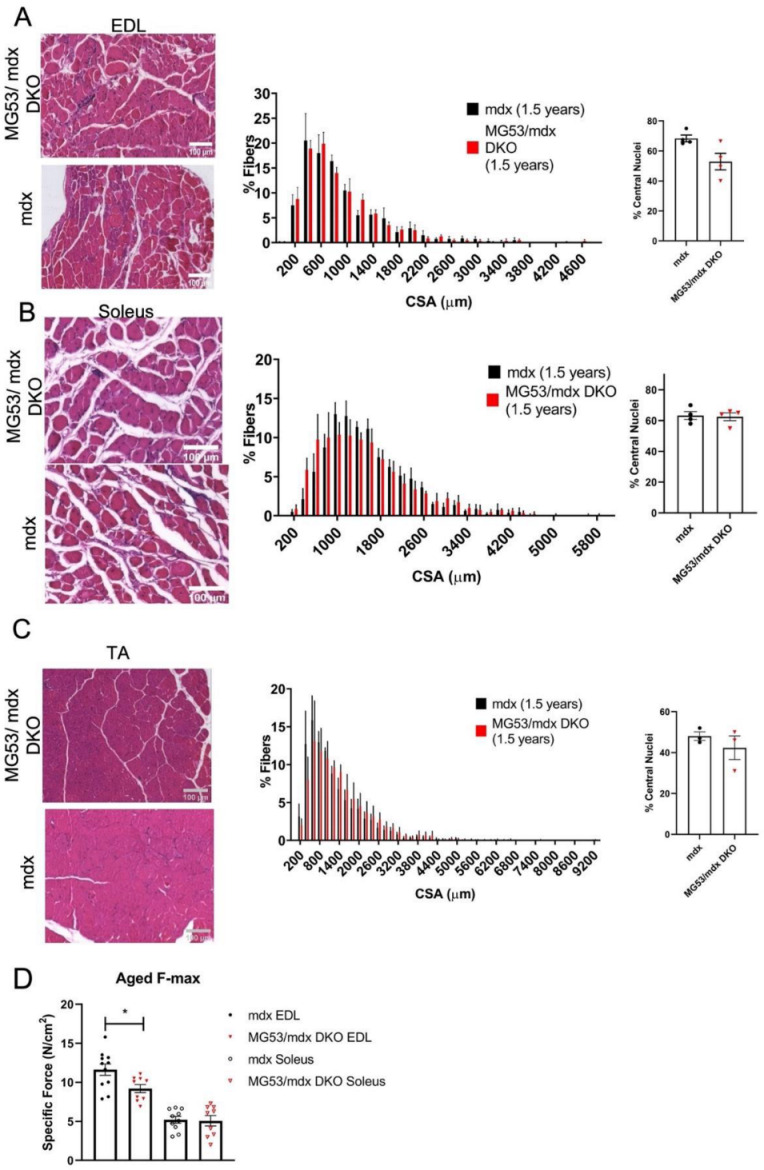
Histology and functional analysis of skeletal muscle of *mdx* and DKO mice at 1.5 years. H&E staining of EDL (**A**), soleus (**B**), TA (**C**) sections from *mdx* and DKO mice at 1.5 years of age was analyzed for frequency distribution of myocyte cross-sectional area (CSA) and central nuclei, *n* = 4 per group (**D**). Maximal force of ex vivo EDL and soleus muscles from *mdx* and DKO mice. EDL *mdx n* = 11, DKO *n* = 9, *p* = 0.0150; Soleus *mdx n* = 10, DKO *n* = 9, *p* = 0.8944. CSA was analyzed with two-way ANOVA. Differences in central nuclei counts and maximal force were tested for statistical significance with an unpaired two-tailed *t*-test assuming unequal variances, and with Welch’s *t*-test for unbalanced designs. * = *p* < 0.05. Data represented as means ± SEM. Scale bars = 100 µm.

Since there was no major increase in structural defects in the young DKO mouse muscle, we aged these animals to determine if the accumulation of membrane injuries over time would lead to a difference in pathology between the *mdx* and DKO mice. This is especially relevant in mice that have compromised membrane repair and membrane fragility because an age-dependent phenotype was observed in the dysferlin-deficient mouse [60]. Additionally, previous studies indicate that TRIM72/MG53 levels of expression decrease in the aging mouse heart [66]. We tested the levels of TRIM72/MG53 in the soleus and EDL muscles of the young and aged *mdx* mice to determine if there were changes in the expression levels with age. This showed that TRIM72/MG53 protein levels dropped in the aged EDL, while there was no change in the soleus muscle (Supplemental Figure S1). We observed that the DKO mice developed the stereotypical hunched posture of the *mdx* mouse by 1.5 years of age and showed significantly decreased body mass compared to *mdx* mice (*mdx*: 32.0 g ± 0.9, *n* = 6 DKO: 27.9 g ± 1.1, *n* = 7, *p* = 0.0161). These differences in body mass could result from metabolic differences in the TRIM72/MG53 knockout mouse background [20,67]. However, the masses of individual skeletal muscles, the heart, and the diaphragm were not significantly different between the *mdx* and DKO mice when the muscles were normalized to their decreased body weight at this age (data not shown). Histologically, there was also no difference in cross-sectional area or central nuclei counts in the EDL, soleus, and TA muscles (Figure 4A–C). We did observe that the force generated from both mouse lines decreased when compared to the younger 6-week-old mice, as expected due to the progression of the DMD phenotype. Interestingly, the EDL exhibited significantly less force in the DKO mouse when compared to the *mdx* mouse while the soleus performed the same in each mouse line, just as we observed in the 6-week-old mice (Figure 4D).

### 3.5. Increased Fibrosis in the Striated Muscle of Aged DKO Mice

**Figure 5:**
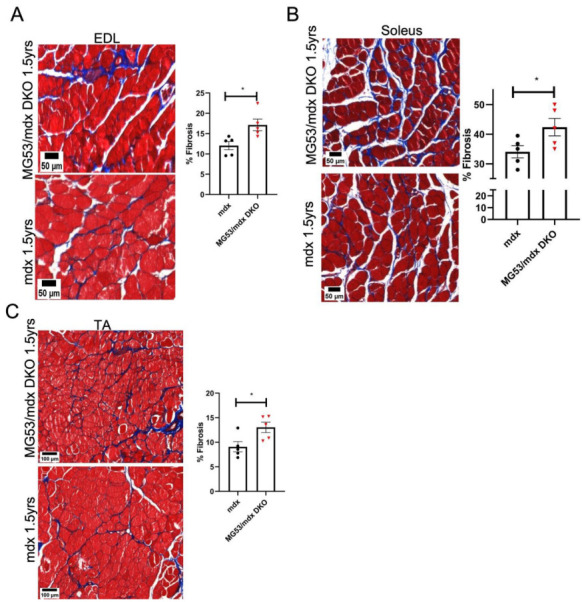
Masson’s trichrome stain analysis implicates robust fibrosis of DKO mice at 1.5 years. DKO mice experience repeated bouts of damage throughout their lifetime, leading to increased fibrotic tissue in skeletal (**A**–**C**) muscle. Data represented as means ± SEM. *n* = 5 for all groups. EDL *p* = 0.0216, Soleus *p* = 0.0490, TA *p* = 0.0280. * = *p* < 0.05. (**A**,**B**) Scale bar = 50 µm, (**C**) Scale bar = 100 µm.

The repeated damage to muscle that occurs during the progression of muscular dystrophy eventually results in the extensive deposition of fibrous tissue, which impairs muscle function and myocyte regeneration, increasing susceptibility to re-injury [68]. The *mdx* mouse undergoes repeated cycles of muscle damage and regeneration, which results in increased fibrotic muscle tissue as the mice age [63], and this phenotype is accelerated in the DKO mouse. At 1.5 years of age, the DKO mice display significantly more fibrotic tissue in every tested skeletal muscle (soleus, EDL, and TA) as compared to the *mdx* mouse (Figure 5A–C).

### 3.6. Sarcolemmal Membrane Integrity and Repair Is Compromised in Aged DKO Mice

**Figure 6:**
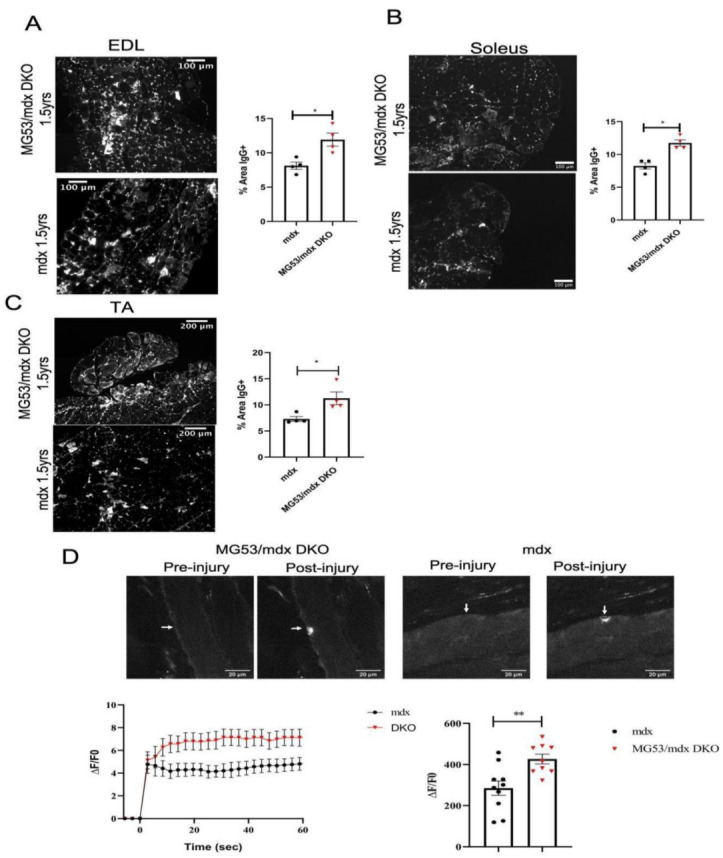
Membrane repair and integrity is compromised in aged DKO mice. (**A**) Representative images of IgG staining. Paraffin sections of EDL, soleus, and TA muscle stained with fluorescent anti-mouse-IgG antibodies demonstrate the distribution of IgG-positive and IgG-negative myocytes in selected skeletal muscles. Quantification analysis of IgG-positive myocytes for the EDL, soleus, and TA show a significant increase in positive muscle myocyte damage in the DKO group. Data analyzed by unpaired two-tailed *t*-test assuming unequal variances, with Welch’s *t*-test for unbalanced designs. *n* = 4 for all groups, EDL *p* = 0.0192, Soleus *p* = 0.0221, TA *p* = 0.0399. (**B**) Representative images and time-dependent accumulation of FM4-64 dye in whole FDB muscles from *mdx* and DKO mice. Laser injury was induced at time 0 on the time course graph. The area under the curve (AUC) of FM4-64 fluorescence traces displays different membrane resealing in DKO mice. AUC was analyzed with a *t*-test. *mdx n* = 10, DKO *n* = 9, *p* = 0.0046. * = *p* < 0.05, ** = *p* < 0.01. Data represented as means ± SEM, (**A**,**B**), Scale bar = 100 µm, (**C**) Scale bar = 200 µm, (**D**) Scale bar = 20 µm.

Aged muscle undergoes various molecular and physical changes in response to the stresses of aging. This is particularly relevant to dystrophic muscle due to repeated bouts of injury and regeneration. Similar to the fibrosis analysis, the DKO EDL, soleus, and TA muscles all showed breakdown of membrane integrity when compared to aged *mdx* mice by IgG staining (Figure 6A–C). The DKO mice also had decreased membrane repair when tested with a laser injury assay (Figure 6D). These results confirm the value of membrane repair in the *mdx* model. These results indicate that loss of TRIM72/MG53-mediated membrane repair, although not debilitating in young mice, is relevant for the maintenance of myocytes as they undergo repeated bouts of damage as the mice age.

## 4. Discussion

DMD myocytes are dependent on the membrane repair process due to the lack of the dystrophin protein that provides a force-transferring link from the cytoskeleton to the extracellular matrix. Our studies show that myocytes from *mdx* dystrophic muscles are dependent on the TRIM72/MG53-mediated membrane repair to survive repeated injury to the sarcolemmal membrane. At six weeks of age, we observed minimal histological differences between the DKO and *mdx* mouse; however, we found that the EDL muscle of the DKO mouse was functionally weaker than that of the *mdx* age-matched control muscles. These changes at a young age also include increased protein expression levels of two TRIM72/MG53 binding partners, dysferlin and caveolin-3, potentially in an attempt to compensate for the absence of TRIM72/MG53. This could occur through clearance of damaged portions of the membrane, reducing inflammatory signaling and limiting collagen deposition [68,69]. 

In response to aging, DKO mice develop significantly more fibrotic tissue, a hallmark of muscular dystrophy, in all skeletal muscles examined. We interpret these findings to indicate that the absence of TRIM72/MG53 exacerbates certain hallmarks of DMD, with these effects being somewhat blunted by the upregulation of other membrane repair proteins or because the absence of TRIM72/MG53 may alter other aspects of muscle function [21]. However, as the DKO animals age, the accumulated membrane damage may overwhelm these compensatory mechanisms in some muscles, causing the animals to develop more extensive fibrosis in their striated muscle tissue. In other muscles, this compromised membrane repair was insufficient to exacerbate the extensive muscle pathology observed in the *mdx* mice. Since the *mdx* mouse shows high levels of muscle pathology at baseline, the decreased membrane repair may not produce a major elevation in pathological measurements, which are already quite high in some muscles. Additionally, various age-related changes could contribute to the observed differences between the young and the old mice. We observed that TRIM72/MG53 protein levels decreased with age in the EDL muscle, while the soleus muscle showed no change (Supplemental Figure S1), which could contribute to some of the differences that we observed comparing one anatomical muscle type to another. Conversely, age-related muscle wasting may not be a major factor as previous studies indicate that there is limited sarcopenia in the hind limb muscles of strain-matched mice at 1.5 years of age [70].

These results indicate that membrane repair is an important process for DMD myocytes to avoid death due to membrane fragility and support that targeting membrane repair could be an effective therapeutic approach to treat DMD. Our findings point to the value of membrane repair in compensating for the membrane fragility observed in DMD, supporting previous studies that illustrated the importance of membrane repair in dystrophic skeletal muscle [60]. Deletion of the TRIM72/MG53 binding partner dysferlin in the *mdx* model also leads to increased myocyte necrosis and muscle weakness. This mouse model was observed to have increased muscle damage at 6 months of age when compared to the *mdx* mouse [60]. Although both dysferlin and TRIM72/MG53 play a role in membrane repair, dysferlin is also involved in satellite cell fusion [71], whereas TRIM72/MG53 is primarily expressed in adult myocytes and mature myotubes [13]. The formation of new myotubes is crucially important in *mdx* mice due to its susceptibility to membrane damage and is likely a cause of differences between these double knockout models. The combination of these results indicates that the loss of any one of these proteins can compromise membrane repair and accelerate the dystrophic phenotype. Future studies assessing the combinatorial loss of membrane repair proteins in dystrophic muscle will help to establish the relative contribution of each protein and how compensatory changes in the expression of membrane repair proteins can contribute to the phenotype seen in the *mdx* mouse.

Our results also indicate that TRIM72/MG53 is important for the function of the EDL muscle of dystrophic mice. EDL muscles from 6-week- and 1.5-year-old DKO mice generate less maximal contractile force when compared to *mdx* age-matched controls. Previous studies have shown that the EDL of *mdx* mice is particularly susceptible to contraction-related membrane injuries [72]. As such, the fast twitch nature of the EDL muscle may increase the strain on the membrane caused by the muscle contraction, leading to muscle damage and a drop in force. We speculate that this membrane damage may not be sufficient to produce additional overt muscle fiber death, as the formation of transient disruptions of the sarcolemmal membrane could lead to increased CK release and IgG entry; however, a sufficient number of muscle fibers are still capable of surviving these disruptions so that this does not result in additional death of the muscle fibers. In this scenario, the drop in force may be due to the fiber type or mechanical effects on the muscles themselves, such as the increased susceptibility of the *mdx* EDL to contraction-related membrane injuries [72].

Decreased force production in the EDL muscle also argues against a role for increased caveolin-3 and dysferlin fully compensating for the loss of TRIM72/MG53 expression. Our previous work indicates that TRIM72/MG53 plays more of a role in the function of dysferlin [61] than it does for caveolins and related caveolar proteins [15]. This increased expression is observed in the EDL muscle and it does not prevent this decline in contractile force production, supporting that TRIM72/MG53 functions not directly related to membrane repair [64,65] could contribute to this difference in the muscle of the two mouse lines. Additional functions of TRIM72/MG53 could also contribute to the decreased body weight seen in the aged DKO animals since previous reports link TRIM72/MG53 function to metabolism [73]. It is also possible that TRIM72/MG53 could be affecting multiple steps in the membrane repair processes. In these studies, we assessed the immediate membrane resealing process necessary to rapidly restore the membrane barrier function. For effective membrane repair to allow cell survival, this resealing must be followed by remodeling of the membrane and other cell components at the injury site to restore normal cell function [74,75,76]. How TRIM72/MG53 functions in these later remodeling steps is unclear and will be a subject of further investigation.

There are also certain limitations to our studies. In certain experimental circumstances, we could not collect all endpoints from certain muscle types. For example, it is challenging to collect contractility measurements of the tibialis anterior muscle using our ex vivo approach. In other experiments, biochemical or histological measurements were performed on muscles that were not used for contractility measurements to avoid confounding changes that could occur to muscles during ex vivo contractility assessments. It is not uncommon for specific anatomical muscles to be more affected than others in patients with certain neuromuscular diseases. This is the case in DMD patients and the *mdx* mouse, where the diaphragm is heavily affected while the heart is relatively spared. Different anatomical muscles show varying levels of fibrosis in the *mdx* mouse (Figure 5) and divergent responses in their TRIM72/MG53 expression levels with aging (Supplemental Figure S1). This suggests that the responses to injury in various muscles could contribute to the phenotypic differences that we see between anatomical muscles, Thus, care should be taken in comparing results from one muscle to another. It should also be noted that there are several factors that could contribute to the changes in the number of IgG-positive fibers that we observe in the aged animals. The number of IgG-positive fibers reflects the number of muscle fibers where there is a loss of sarcolemmal membrane barrier function, which could result from increased fragility of the fibers with age, even in the absence of an overt change in membrane repair capacity.

## 5. Conclusions

Our current study focused on the value of membrane repair in the *mdx* mouse model of DMD. We knocked out the membrane repair protein TRIM72/MG53 to elicit a membrane repair defect in these mice. Minimal histological changes were observed in 6-week-old mice; however, robust fibrosis and reduced membrane integrity were observed in aged mice. This suggests that the dystrophic muscle can compensate for membrane repair deficits early in life; however, in the absence of membrane repair, the continuous bouts of injury that lead to increased death of the fragile dystrophic myocytes tax the regenerative capacity of muscle, leading to increased fibrosis and loss of muscle function. This model demonstrates the value of membrane repair in repeated injury and supports a role for TRIM72/MG53 in maintaining myocyte survival in dystrophic muscle.

## Data Availability

The datasets produced and analyzed during the current study are available from the corresponding author following a reasonable request.

## Supplementary Materials

The following supporting information can be downloaded at: https://www.mdpi.com/article/10.3390/cells11091417/s1, Figure S1: MG53 expression in young and aged mdx muscle. (A) Soleus and (B) EDL muscle lysates from 6-week (young) and 1.5 years old (aged) mdx mice.

## Abbreviations

area under the curve (AUC); bovine serum albumin (BSA); creatine kinase (CK); cross-sectional area (CSA); double knockout (DKO); Duchenne muscular dystrophy (DMD); enhanced chemiluminescence (ECL); enzyme-linked immunoassay (ELISA); extensor digitorum longus (EDL); flexor digitorum brevis (FDB); horseradish peroxidase (HRP); immunoglobulin G (IgG); maximal force (F-max); mitsugumin 53 (MG53); poloxamer 188 (P188); Radioimmunoprecipitation Assay buffer (RIPA); recombinant human MG53 protein (rhMG53); RING B-box Coiled Coil (RBCC); tibialis anterior (TA); tripartite motif (TRIM).

## References

[1] Bi G.Q., Alderton J.M., Steinhardt R.A. (1995). Calcium-regulated exocytosis is required for cell membrane resealing. J. Cell Biol..

[2] McNeil P.L., Ito S. (1989). Gastrointestinal cell plasma membrane wounding and resealing in vivo. Gastroenterology.

[3] McNeil P.L., Khakee R. (1992). Disruptions of muscle fiber plasma membranes. Role in exercise-induced damage. Am. J. Pathol..

[4] Steinhardt R.A., Bi G., Alderton J.M. (1994). Cell membrane resealing by a vesicular mechanism similar to neurotransmitter release. Science.

[5] Miyake K., McNeil P.L. (1995). Vesicle accumulation and exocytosis at sites of plasma membrane disruption. J. Cell Biol..

[6] Reddy A., Caler E.V., Andrews N.W. (2001). Plasma membrane repair is mediated by Ca^2+^-regulated exocytosis of lysosomes. Cell.

[7] Idone V., Tam C., Goss J.W., Toomre D., Pypaert M., Andrews N.W. (2008). Repair of injured plasma membrane by rapid Ca2+-dependent endocytosis. J. Cell Biol..

[8] Bement W.M., Forscher P., Mooseker M.S. (1993). A novel cytoskeletal structure involved in purse string wound closure and cell polarity maintenance. J. Cell Biol..

[9] Babiychuk E.B., Monastyrskaya K., Potez S., Draeger A. (2009). Intracellular Ca^2+^ operates a switch between repair and lysis of streptolysin O-perforated cells. Cell Death Differ..

[10] Jimenez A.J., Maiuri P., Lafaurie-Janvore J., Divoux S., Piel M., Perez F. (2014). ESCRT machinery is required for plasma membrane repair. Science.

[11] Keyel P.A., Loultcheva L., Roth R., Salter R.D., Watkins S.C., Yokoyama W.M., Heuser J.E. (2011). Streptolysin O clearance through sequestration into blebs that bud passively from the plasma membrane. J. Cell Sci..

[12] Friden J., Sjostrom M., Ekblom B. (1983). Myofibrillar damage following intense eccentric exercise in man. Int. J. Sports Med..

[13] Lin P., Zhu H., Cai C., Wang X., Cao C., Xiao R., Pan Z., Weisleder N., Takeshima H., Ma J. (2012). Nonmuscle myosin IIA facilitates vesicle trafficking for MG53-mediated cell membrane repair. FASEB J..

[14] Weisleder N., Takeshima H., Ma J. (2009). Mitsugumin 53 (MG53) facilitates vesicle trafficking in striated muscle to contribute to cell membrane repair. Commun. Integr. Biol..

[15] Cai C., Weisleder N., Ko J.K., Komazaki S., Sunada Y., Nishi M., Takeshima H., Ma J. (2009). Membrane repair defects in muscular dystrophy are linked to altered interaction between MG53, caveolin-3, and dysferlin. J. Biol. Chem..

[16] Codding S.J., Marty N., Abdullah N., Johnson C.P. (2016). Dysferlin Binds SNAREs (Soluble N-Ethylmaleimide-sensitive Factor (NSF) Attachment Protein Receptors) and Stimulates Membrane Fusion in a Calcium-sensitive Manner. J. Biol. Chem..

[17] Hatakeyama S. (2011). TRIM proteins and cancer. Nat. Rev. Cancer.

[18] Ozato K., Shin D.M., Chang T.H., Morse H.C. (2008). TRIM family proteins and their emerging roles in innate immunity. Nat. Rev. Immunol..

[19] Weisleder N., Takeshima H., Ma J. (2008). Immuno-proteomic approach to excitation--contraction coupling in skeletal and cardiac muscle: Molecular insights revealed by the mitsugumins. Cell Calcium.

[20] Lee C.S., Yi J.S., Jung S.Y., Kim B.W., Lee N.R., Choo H.J., Jang S.Y., Han J., Chi S.G., Park M. (2010). TRIM72 negatively regulates myogenesis via targeting insulin receptor substrate-1. Cell Death Differ..

[21] Lee H., Park J.J., Nguyen N., Park J.S., Hong J., Kim S.H., Song W.Y., Kim H.J., Choi K., Cho S. (2016). MG53-IRS-1 (Mitsugumin 53-Insulin Receptor Substrate-1) Interaction Disruptor Sensitizes Insulin Signaling in Skeletal Muscle. J. Biol. Chem..

[22] Cai C., Masumiya H., Weisleder N., Matsuda N., Nishi M., Hwang M., Ko J.K., Lin P., Thornton A., Zhao X. (2009). MG53 nucleates assembly of cell membrane repair machinery. Nat. Cell Biol..

[23] Cao C.M., Zhang Y., Weisleder N., Ferrante C., Wang X., Lv F., Song R., Hwang M., Jin L., Guo J. (2010). MG53 constitutes a primary determinant of cardiac ischemic preconditioning. Circulation.

[24] Duann P., Li H., Lin P., Tan T., Wang Z., Chen K., Zhou X., Gumpper K., Zhu H., Ludwig T. (2015). MG53-mediated cell membrane repair protects against acute kidney injury. Sci. Transl. Med..

[25] Kim S.C., Kellett T., Wang S., Nishi M., Nagre N., Zhou B., Flodby P., Shilo K., Ghadiali S.N., Takeshima H. (2014). TRIM72 is required for effective repair of alveolar epithelial cell wounding. Am. J. Physiol. Lung Cell Mol. Physiol..

[26] Cai C., Masumiya H., Weisleder N., Pan Z., Nishi M., Komazaki S., Takeshima H., Ma J. (2009). MG53 regulates membrane budding and exocytosis in muscle cells. J. Biol. Chem..

[27] Bansal D., Miyake K., Vogel S.S., Groh S., Chen C.C., Williamson R., McNeil P.L., Campbell K.P. (2003). Defective membrane repair in dysferlin-deficient muscular dystrophy. Nature.

[28] Defour A., Van der Meulen J.H., Bhat R., Bigot A., Bashir R., Nagaraju K., Jaiswal J.K. (2014). Dysferlin regulates cell membrane repair by facilitating injury-triggered acid sphingomyelinase secretion. Cell Death Dis..

[29] Lennon N.J., Kho A., Bacskai B.J., Perlmutter S.L., Hyman B.T., Brown R.H. (2003). Dysferlin interacts with annexins A1 and A2 and mediates sarcolemmal wound-healing. J. Biol. Chem..

[30] Bouter A., Gounou C., Bérat R., Tan S., Gallois B., Granier T., d’Estaintot B.L., Pöschl E., Brachvogel B., Brisson A.R. (2011). Annexin-A5 assembled into two-dimensional arrays promotes cell membrane repair. Nat. Commun..

[31] Demonbreun A.R., Quattrocelli M., Barefield D.Y., Allen M.V., Swanson K.E., McNally E.M. (2016). An actin-dependent annexin complex mediates plasma membrane repair in muscle. J. Cell Biol..

[32] McNeil A.K., Rescher U., Gerke V., McNeil P.L. (2006). Requirement for annexin A1 in plasma membrane repair. J. Biol. Chem..

[33] Rezvanpour A., Santamaria-Kisiel L., Shaw G.S. (2011). The S100A10-annexin A2 complex provides a novel asymmetric platform for membrane repair. J. Biol. Chem..

[34] Swaggart K.A., Demonbreun A.R., Vo A.H., Swanson K.E., Kim E.Y., Fahrenbach J.P., Holley-Cuthrell J., Eskin A., Chen Z., Squire K. (2014). Annexin A6 modifies muscular dystrophy by mediating sarcolemmal repair. Proc. Natl. Acad. Sci. USA.

[35] Corrotte M., Almeida P.E., Tam C., Castro-Gomes T., Fernandes M.C., Millis B.A., Cortez M., Miller H., Song W., Maugel T.K. (2013). Caveolae internalization repairs wounded cells and muscle fibers. elife.

[36] Hernández-Deviez D.J., Howes M.T., Laval S.H., Bushby K., Hancock J.F., Parton R.G. (2008). Caveolin regulates endocytosis of the muscle repair protein, dysferlin. J. Biol. Chem..

[37] Tagawa K., Ogawa M., Kawabe K., Yamanaka G., Matsumura T., Goto K., Nonaka I., Nishino I., Hayashi Y.K. (2003). Protein and gene analyses of dysferlinopathy in a large group of Japanese muscular dystrophy patients. J. Neurol. Sci..

[38] Fanin M., Angelini C. (2002). Muscle pathology in dysferlin deficiency. Neuropathol. Appl. NeuroBiol..

[39] Mahjneh I., Marconi G., Bushby K., Anderson L.V., Tolvanen-Mahjneh H., Somer H. (2001). Dysferlinopathy (LGMD2B): A 23-year follow-up study of 10 patients homozygous for the same frameshifting dysferlin mutations. Neuromuscul. Disord..

[40] Dincer P., Akcoren Z., Demir E., Richard I., Sancak O., Kale G., Ozme S., Karaduman A., Tan E., Urtizberea J.A. (2000). A cross section of autosomal recessive limb-girdle muscular dystrophies in 38 families. J. Med. Genet..

[41] Galbiati F., Volonte D., Minetti C., Chu J.B., Lisanti M.P. (1999). Phenotypic behavior of caveolin-3 mutations that cause autosomal dominant limb girdle muscular dystrophy (LGMD-1C). Retention of LGMD-1C caveolin-3 mutants within the golgi complex. J. Biol. Chem..

[42] Minetti C., Sotgia F., Bruno C., Scartezzini P., Broda P., Bado M., Masetti E., Mazzocco M., Egeo A., Donati M.A. (1998). Mutations in the caveolin-3 gene cause autosomal dominant limb-girdle muscular dystrophy. Nat. Genet..

[43] Waddell L.B., Lemckert F.A., Zheng X.F., Tran J., Evesson F.J., Hawkes J.M., Lek A., Street N.E., Lin P., Clarke N.F. (2011). Dysferlin, annexin A1, and mitsugumin 53 are upregulated in muscular dystrophy and localize to longitudinal tubules of the T-system with stretch. J. Neuropathol. Exp. Neurol..

[44] Corrotte M., Fernandes M.C., Tam C., Andrews N.W. (2012). Toxin pores endocytosed during plasma membrane repair traffic into the lumen of MVBs for degradation. Traffic.

[45] Gu J.H., Ge J.B., Li M., Xu H.D., Wu F., Qin Z.H. (2013). Poloxamer 188 protects neurons against ischemia/reperfusion injury through preserving integrity of cell membranes and blood brain barrier. PLoS ONE.

[46] Yao Y., Zhang B., Zhu H., Li H., Han Y., Chen K., Wang Z., Zeng J., Liu Y., Wang X. (2016). MG53 permeates through blood-brain barrier to protect ischemic brain injury. Oncotarget.

[47] Liu J., Zhu H., Zheng Y., Xu Z., Li L., Tan T., Park K.H., Hou J., Zhang C., Li D. (2015). Cardioprotection of recombinant human MG53 protein in a porcine model of ischemia and reperfusion injury. J. Mol. Cell. Cardiol..

[48] Zhu H., Hou J., Roe J.L., Park K.H., Tan T., Zheng Y., Li L., Zhang C., Liu J., Liu Z. (2015). Amelioration of ischemia-reperfusion-induced muscle injury by the recombinant human MG53 protein. Muscle Nerve.

[49] Liu J., Aoki M., Illa I., Wu C., Fardeau M., Angelini C., Serrano C., Urtizberea J.A., Hentati F., Hamida M.B. (1998). Dysferlin, a novel skeletal muscle gene, is mutated in Miyoshi myopathy and limb girdle muscular dystrophy. Nat. Genet..

[50] Ryder S., Leadley R.M., Armstrong N., Westwood M., de Kock S., Butt T., Jain M., Kleijnen J. (2017). The burden, epidemiology, costs and treatment for Duchenne muscular dystrophy: An evidence review. Orphanet J. Rare Dis..

[51] Mendell J.R., Lloyd-Puryear M. (2013). Report of MDA muscle disease symposium on newborn screening for Duchenne muscular dystrophy. Muscle Nerve.

[52] Weisleder N., Takizawa N., Lin P., Wang X., Cao C., Zhang Y., Tan T., Ferrante C., Zhu H., Chen P.J. (2012). Recombinant MG53 protein modulates therapeutic cell membrane repair in treatment of muscular dystrophy. Sci. Transl. Med..

[53] Markham B.E., Kernodle S., Nemzek J., Wilkinson J.E., Sigler R. (2015). Chronic Dosing with Membrane Sealant Poloxamer 188 NF Improves Respiratory Dysfunction in Dystrophic Mdx and Mdx/Utrophin-/- Mice. PLoS ONE.

[54] Houang E.M., Haman K.J., Filareto A., Perlingeiro R.C., Bates F.S., Lowe D.A., Metzger J.M. (2015). Membrane-stabilizing copolymers confer marked protection to dystrophic skeletal muscle in vivo. Mol. Ther. Methods Clin. Dev..

[55] Renzini A., Marroncelli N., Cavioli G., Di Francescantonio S., Forcina L., Lambridis A., Di Giorgio E., Valente S., Mai A., Brancolini C. (2022). Cytoplasmic HDAC4 regulates the membrane repair mechanism in Duchenne muscular dystrophy. J. Cachexia Sarcopenia Muscle.

[56] Mâncio R., Hermes T., Macedo A., Mizobuti D., Valduga A., Rupcic I., Minatel E. (2017). Vitamin E treatment decreases muscle injury in mdx mice. Nutrition.

[57] Quattrocelli M., Salamone I., Page P., Warner J., Demonbreun A., McNally E. (2017). Intermittent Glucocorticoid Dosing Improves Muscle Repair and Function in Mice with Limb-Girdle Muscular Dystrophy. Am. J. Pathol..

[58] Vila M., Rayavarapu S., Hogarth M., Van der Meulen J., Horn A., Defour A., Takeda S., Brown K., Hathout Y., Nagaraju K. (2017). Mitochondria mediate cell membrane repair and contribute to Duchenne muscular dystrophy. Cell Death Differ..

[59] Wright P., McKinney E., Berry S., Evers A., Kent C. (1984). A functional membrane repair system in Duchenne muscular dystrophy fibroblasts. J. Neurol. Sci..

[60] Han R., Rader E.P., Levy J.R., Bansal D., Campbell K.P. (2011). Dystrophin deficiency exacerbates skeletal muscle pathology in dysferlin-null mice. Skelet Muscle.

[61] Matsuda C., Miyake K., Kameyama K., Keduka E., Takeshima H., Imamura T., Araki N., Nishino I., Hayashi Y. (2012). The C2A domain in dysferlin is important for association with MG53 (TRIM72). PLoS Curr..

[62] Radley-Crabb H.G., Marini J.C., Sosa H.A., Castillo L.I., Grounds M.D., Fiorotto M.L. (2014). Dystropathology increases energy expenditure and protein turnover in the mdx mouse model of duchenne muscular dystrophy. PLoS ONE.

[63] Dupont-Versteegden E.E., McCarter R.J. (1992). Differential expression of muscular dystrophy in diaphragm versus hindlimb muscles of mdx mice. Muscle Nerve.

[64] Motohashi N., Alexander M.S., Shimizu-Motohashi Y., Myers J.A., Kawahara G., Kunkel L.M. (2013). Regulation of IRS1/Akt insulin signaling by microRNA-128a during myogenesis. J. Cell Sci..

[65] Yi J.-S., Park J.S., Ham Y.-M., Nguyen N., Lee N.-R., Hong J., Kim B.-W., Lee H., Lee C.-S., Jeong B.-C. (2013). MG53-induced IRS-1 ubiquitination negatively regulates skeletal myogenesis and insulin signalling. Nat. Commun..

[66] Wang X., Li X., Ong H., Tan T., Park K., Bian Z., Zou X., Haggard E., Janssen P., Merritt R. (2021). MG53 suppresses NF-κB activation to mitigate age-related heart failure. JCI Insight.

[67] Jiang P., Ren L., Zhi L., Yu Z., Lv F., Xu F., Peng W., Bai X., Cheng K., Quan L. (2020). Negative regulation of AMPK signaling by high glucose via E3 ubiquitin ligase MG53. Mol. Cell.

[68] Wynn T.A. (2008). Cellular and molecular mechanisms of fibrosis. J. Pathol..

[69] Tidball J.G., Villalta S.A. (2010). Regulatory interactions between muscle and the immune system during muscle regeneration. Am. J. Physiol. Regul. Integr. Comp. Physiol..

[70] Hamrick M., Ding K., Pennington C., Chao Y., Wu Y., Howard B., Immel D., Borlongan C., McNeil P., Bollag W. (2006). Age-related loss of muscle mass and bone strength in mice is associated with a decline in physical activity and serum leptin. Bone.

[71] Cohen T.V., Cohen J.E., Partridge T.A. (2012). Myogenesis in dysferlin-deficient myoblasts is inhibited by an intrinsic inflammatory response. Neuromuscul. Disord..

[72] Moens P., Baatsen P.H., Marechal G. (1993). Increased susceptibility of EDL muscles from mdx mice to damage induced by contractions with stretch. J. Muscle Res. Cell Motil..

[73] Wu H.K., Zhang Y., Cao C.M., Hu X., Fang M., Yao Y., Jin L., Chen G., Jiang P., Zhang S. (2019). Glucose-Sensitive Myokine/Cardiokine MG53 Regulates Systemic Insulin Response and Metabolic Homeostasis. Circulation.

[74] Huang Y., de Morrée A., van Remoortere A., Bushby K., Frants R.R., den Dunnen J.T., van der Maarel S.M. (2008). Calpain 3 is a modulator of the dysferlin protein complex in skeletal muscle. Hum. Mol. Genet..

[75] Duguez S., Bartoli M., Richard I. (2006). Calpain 3: A key regulator of the sarcomere?. FEBS J..

[76] Mellgren R.L., Miyake K., Kramerova I., Spencer M.J., Bourg N., Bartoli M., Richard I., Greer P.A., McNeil P.L. (2009). Calcium-dependent plasma membrane repair requires m- or mu-calpain, but not calpain-3, the proteasome, or caspases. Biochim. Biophys. Acta.

