# Supplementary material for: Reduced Sarcolemmal Membrane Repair Exacerbates Striated Muscle Pathology in a Mouse Model of Duchenne Muscular Dystrophy
Source: Cells. 2022 Apr 22;11(9):1417. doi: 10.3390/cells11091417 (PMC9100510; doi:10.3390/cells11091417)
Supplement: Supplementary file 1 [file cells-11-01417-s001.zip › cells-1635975 Supplemental Figure S1.pdf]

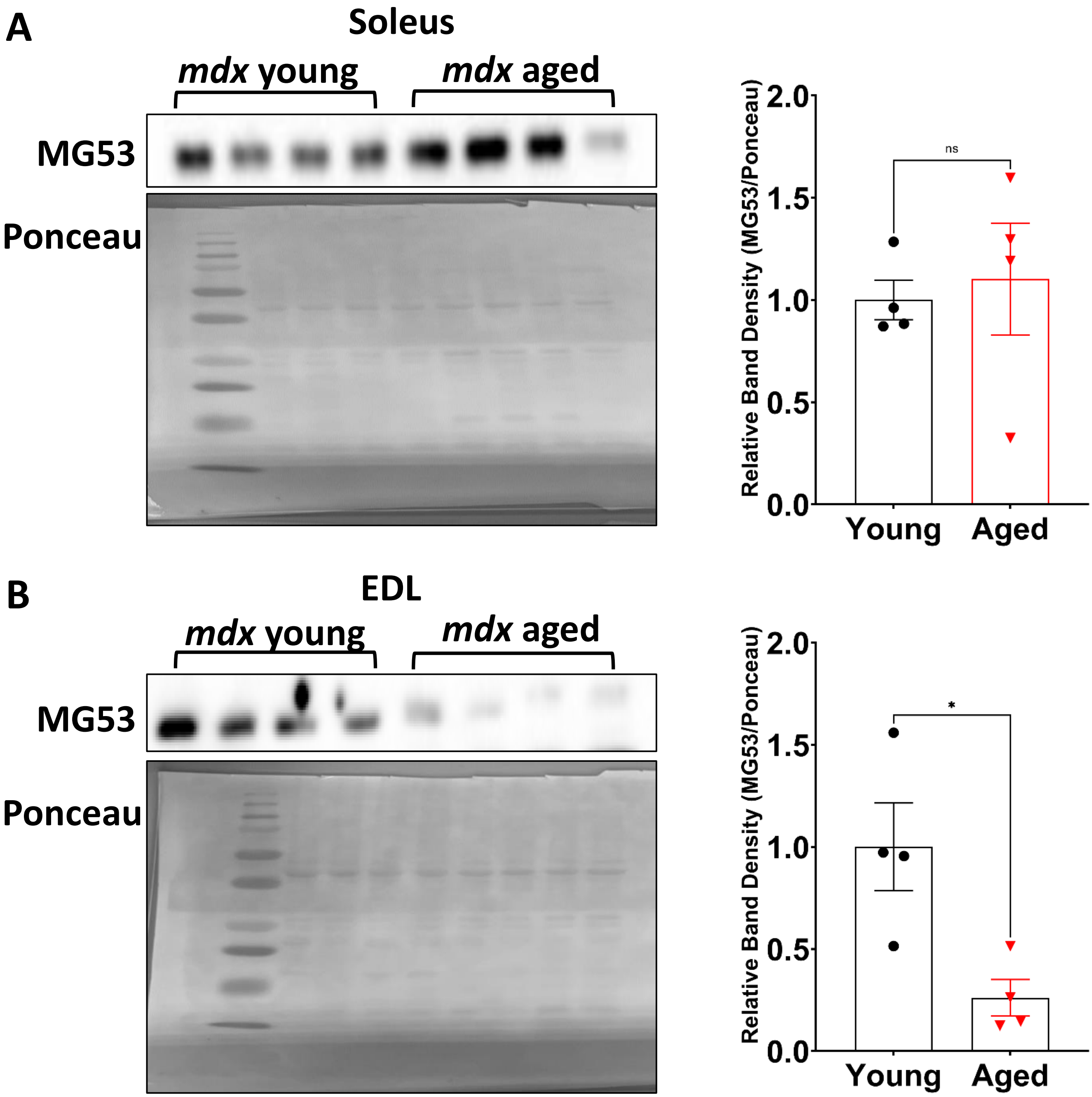

Supplemental Figure S1. MG53 expression in young and aged *mdx* muscle. **(A)** Soleus and **(B)** EDL muscle lysates from 6-week (young) and 1.5 years old (aged) *mdx* mice. MG53 is downregulated in the EDL of aged *mdx* mice as compared to the young, but this remains unchanged in the soleus. Statistical analysis was performed using an unpaired two-tailed t-test assuming unequal variances, Welch's t-test for unbalanced designs. A) (n=4) $p = 0.7376$, B)(n=4) $p = 0.0190$; * = $p < 0.05$. Data is represented as mean $\pm$ SEM.
